# Air pollution and trajectories of adolescent conduct problems: the roles of ethnicity and racism; evidence from the DASH longitudinal study

**DOI:** 10.1007/s00127-021-02097-7

**Published:** 2021-04-30

**Authors:** A. Karamanos, I. Mudway, F. Kelly, S. D. Beevers, D. Dajnak, C. Elia, J. K. Cruickshank, Y. Lu, S. Tandon, E. Enayat, P. Dazzan, M. Maynard, S. Harding

**Affiliations:** 1 Department of Nutritional Sciences, School of Life Course Sciences, Faculty of Life Sciences & Medicine, King’s College London, 57 Waterloo Road, London, SE1 8WA UK; 2 MRC Centre for Environment and Health, Imperial College London, London, UK; 3 NIHR Health Protection Research Unit in Environmental Exposures and Health, Imperial College London, London, UK; 4 Department of Population Health Sciences, School of Population Health & Environmental Sciences, Faculty of Life Sciences & Medicine, King’s College London, London, UK; 5 Division of Psychiatry, Faculty of Brain Sciences, University College London, London, UK; 6 Institute of Psychiatry, Psychology and Neuroscience, King’s College London, London, UK; 7 School of Clinical and Applied Sciences, Leeds Beckett University, London, UK

**Keywords:** Air pollution, Conduct problems, Ethnicity, Racism, Cohort study

## Abstract

**Purpose:**

No known UK empirical research has investigated prospective associations between ambient air pollutants and conduct problems in adolescence. Ethnic minority children are disproportionately exposed to structural factors that could moderate any observed relationships. This prospective study examined whether exposure to PM_2.5_ and NO_2_ concentrations is associated with conduct problems in adolescence, and whether racism or ethnicity moderate such associations.

**Methods:**

Longitudinal associations between annual mean estimated PM_2.5_ and NO_2_ concentrations at the residential address and trajectories of conduct problems, and the potential influence of racism and ethnicity were examined school-based sample of 4775 participants (2002–2003 to 2005–2006) in London, using growth curve models.

**Results:**

Overall, in the fully adjusted model, exposure to lower concentrations of PM_2.5_ and NO_2_ was associated with a decrease in conduct problems during adolescence, while exposure to higher concentrations was associated with a flattened trajectory of conduct symptoms. Racism amplified the effect of PM_2.5_ (*β* = 0.05 (95% CI 0.01 to 0.10, *p* < 0.01)) on adolescent trajectories of conduct problems over time. At higher concentrations of PM_2.5_, there was a divergence of trajectories of adolescent conduct problems between ethnic minority groups, with White British and Black Caribbean adolescents experiencing an increase in conduct problems over time.

**Conclusion:**

These findings suggest that the intersections between air pollution, ethnicity, and racism are important influences on the development of conduct problems in adolescence.

**Supplementary Information:**

The online version contains supplementary material available at 10.1007/s00127-021-02097-7.

## Introduction

Conduct problems refer to behaviours under the conduct-oppositional spectrum, including those that are defiant, antisocial, and/or potentially harmful to others such as lying, stealing, physical aggression, and rule-breaking [1], currently estimated to affect about one in twenty (6.2%) of 11 to 16 years old. Literature shows that, in general, they are higher in childhood and decrease with time [2], while an increasing number of studies indicate that there may be subgroups of conduct behaviours that characterise the heterogeneity in developmental pathways of conduct problem behaviours across childhood and adolescence [3, 4]. They can be markers for co-morbid mental health problems [5], lower educational attainment [6], later criminality [7, 8], and adverse labour market experiences [9–11].

Growing evidence shows strong associations between structural factors such as racial discrimination and neighbourhood deprivation and conduct problems in childhood and adolescence [12–14]. Prior studies also show a link between conduct problems, and harsh discipline, poor parental monitoring, deviant peer affiliation, poverty, parental divorce, and parental psychopathology [15]. Air pollution, as a marker of environmental adversity, can affect adolescent mental health problems [16–20], via possible direct effects on brain neuroinflammation [21, 22], oxidative stress [23], microglial activation [24], cerebrovascular dysfunction, or changes in the blood–brain barrier [25]. Most studies are cross-sectional, with potential selection bias, and only one study modelled both ambient air pollution exposure and development of conduct problems over time [20]. In addition, there has been little attempt to consider gender or age effects, except for a US longitudinal study which reported no effect modification by age [20]. Psychosocial stress induced by experiences of racism may also contribute to differences in adolescent conduct problems either as a direct cause or via enhancing neuroimmune and hypersensitivity response to air pollution [26].

**Fig. 1:**
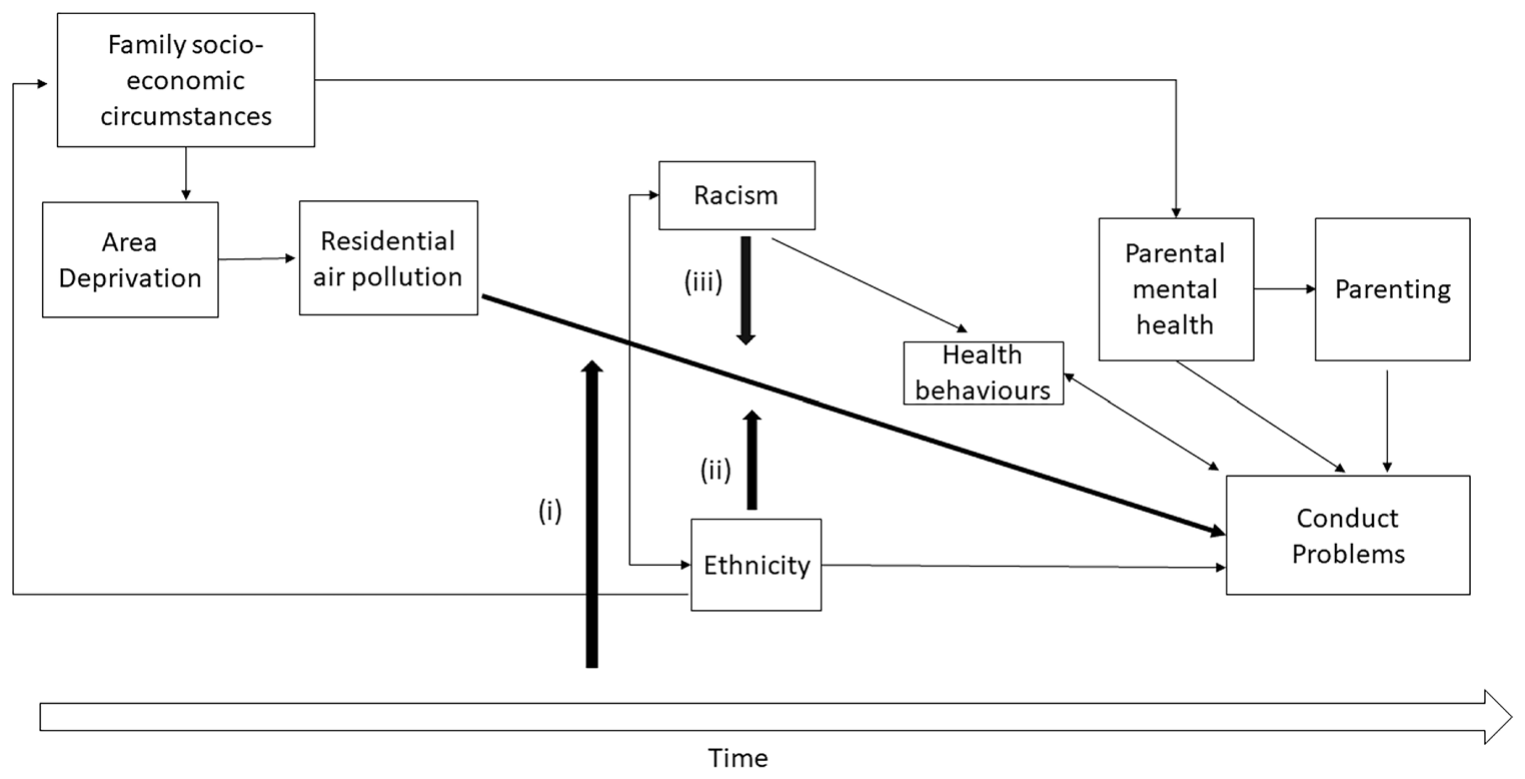
Conceptual framework of the association between residential air pollution and conduct problems

Some ethnic minority groups show better or similar levels of mental health overall in adolescence than White children and adolescents [27] which is not explained by socio-economic circumstances, family type, social support, or perceived parenting [28–30]. Indian, Black African, Pakistani, and Bangladeshi children report fewer behavioural problems, while mixed White/Black Caribbean children report more compared with White children. Ethnic minority adolescents are more likely to report racism, and reside in urban areas where ambient air pollution levels are higher [31]. These areas are also more income-deprived neighbourhoods where unemployment and crime rates are high and could disproportionately affect mental health outcomes [32–34]. No study exists which adequately accounts for intersections between ethnicity and air pollution or air pollution and racism, and their contribution to adolescent mental health. By exploiting the longitudinal design of the DASH study and data from 4775 adolescents aged 11–16 years, we explored the interrelationships between ambient air pollution, time, ethnicity, racism, and conduct problems. Figure 1 shows conceptual framework which guided the analyses of this study. We hypothesised that higher concentrations of fine particulate matter (PM_2.5_) and nitrogen dioxide (NO_2_) would be associated with: (i) a slower decrease of conduct problems over time, (ii) an increase in ethnic differences in conduct problems during adolescence, and (iii) racism would associate with an amplification any observed effect of air pollution on conduct problems.

## Methods

### Design and sample

The DASH longitudinal study is detailed elsewhere [35]. In brief, in 2002–2003, a total of 6631 students, aged 11–13 years, from 51 secondary schools in 10 London boroughs (highlighted in Supplementary Figure 1), took part at baseline. In 2005–2006, 4775 students took part in the follow-up survey. The overall response rate among those invited to take part in the follow-up was 88%. Written informed consent was obtained from all participants.

### Measures

#### Outcome: conduct problems

Conduct problems in adolescence were measured by the conduct problems sub-scale within the self-reported Strengths and Difficulties Questionnaire (SDQ) [36]. Components of the sub-scale relate to whether respondents often had tantrums or hot tempers, were generally obedient, often fought with other children, often lied, or cheated and whether they stole from home, school or elsewhere. ‘Somewhat True’ was scored as 1, but the scoring of ‘Not True’ and ‘Certainly True’ varied with the item (either a score of 0 or 2). A composite conduct problem score was derived with higher scores indicating higher conduct problems.

#### Exposure: residential air pollutant assessments

Modelled annual mean NO_2_, Ozone (O_3_), PM_10_, and PM_2.5_ concentrations for London covering the years 2003–2004 and 2005–2006 were estimated, at a 20 m × 20 m regular grid across the study area, using the King’s College London (KCL) urban dispersion model [37]. The KCL urban model uses Atmospheric Dispersion Modelling System (ADMS) model v4 and road source model v2.3 (CERC19), hourly measured meteorological data, empirically derived NO–NO_2_–O_3_ and PM relationships, and emissions from the London Atmospheric Emissions Inventory (the LAEI;[38]) (the pollutant surface maps for London covering 2003 and 2006 are presented in Supplementary Figure 1). Each yearly model reflected a range of pollutant sources and emissions, including major and minor roads, with detailed information on vehicle stock, traffic flows, and speed on a link-by-link basis. Other sources within the model included large and small regulated industrial processes, boiler plants, domestic and commercial combustion sources, agriculture, rail, ships, airports, and pollution carried into the area by prevailing winds. The model performed well when evaluated against measurements, with low normalised mean bias and high spearman correlation coefficients (ρ) between observed versus modelled concentrations: ρ > 0.9 for NOx, O_3_, PM_10_, and PM_2.5_ and ρ > 0.83 for NO_2_ at both roadside and background locations. Further detail about the modelling procedure and model evaluation has been published previously and is available elsewhere [37, 39]. For linkage to the air pollution data, residential address at the time of survey was geocoded, locating the address to the nearest meter, and assigned annual air pollutant concentrations to the nearest 20 m × 20 m grid point using bilinear interpolation.

Supplementary Figure 2 shows some small improvement in the modelled exposures to NO_2_ and PM_2.5_ between 11–13 and 14–16 years. Average ambient pollution concentrations during follow-up for NO_2_ were 41.6 (range 26.7 to 75.5) µg/m^3^ and PM_2.5_ 20.1 (14.4 to 24.4) µg/m^3^.

#### Effect modifiers: ethnicity and racism

Ethnicity was self-reported, checked against reported parental ethnicity and grandparents’ country of birth. Ethnic groups were categorised as follows; White UK, Black Caribbean, Black African, Indian, Pakistani/Bangladeshi, and Other (Mixed, Irish, Greek, Turkish, Kosovan, Albanian, Kurdish, Vietnamese, Singaporean, Pilipino, Japanese, Chinese, and other Asian).

Reported racism/discrimination (‘no’ or ‘yes’) was assessed at the baseline and follow-up surveys using the experiences of discrimination scale which includes questions on 'unfair treatment' on the grounds of race, skin colour, place of birth, and religion in various locations, e.g., school, work, on the street [40]. These are: ‘Has anyone made you feel bad or hassled you because of your race, skin colour, or where you were born?’ and ‘Has anyone made you feel bad or hassled you because of your religion?’.

### Covariables

A self-complete questionnaire was used to collect information on socio-demographics, parental mental health, and own lifestyle behaviours. Sex as well as parenting style, maternal mental health, adolescent health behaviours, family affluence, family type and labour market status, and neighbourhood deprivation were included in the models as time-variant covariables. Parenting style was captured using the eight-item Parental Bonding Instrument (PBI) which rates each item on a four-point Likert scale from which scores for parental care and parental control are derived [41]. Higher scores represent greater care and greater control. These scores were recoded into tertiles. Maternal mental health was measured by asking participants whether their mother/stepmother had mental health problems at survey time. Health behaviours were assessed by asking participants whether they had ever smoked or drank alcohol.

Family socio-economic circumstances were measured through a family affluence scale and categorised as 0–1 (Least advantaged), 2 (Less advantaged), and ≥ 3 (Least disadvantaged) [42]. Neighbourhood deprivation was measured by the income domain of the Index of Multiple Deprivation in 2004 and 2007 and categorised in quintiles. This area-based deprivation index measures the proportion of population that is living as low-income families at the Lower Super Output geographic level (LSOA). The higher the index score, the higher the area-based deprivation.

### Statistical analysis

To examine the association between air pollution and conduct problems in a sample of 4775 adolescents aged 11–16 years, and whether ethnicity and racism moderate this association, we fitted growth curve models. Preliminary analyses showed no significant clustering of conduct problems within schools or LSOAs.

Growth curve modelling allowed the estimation of individual conduct problem trajectories in adolescence, using age as an independent variable for time. Age (years) was centred at age 12 years (intercept). Analyses used two level models for repeated measures nested in adolescents and one slope for age to account for individual linear conduct problem trajectories.

Statistical analysis was conducted via STATA 16 v.1 (Stat Corp., College Station, Texas, USA). We considered as confounders: (i) factors related to both the exposure and the outcome in bivariable analyses (*P* < 0.20), (ii) factors that modified (> 10% change in regression coefficient) the estimate of the exposure variable, or (iii) factors deemed relevant according to the scientific literature. High correlations were seen between all air pollution concentration estimates (all r's > 0.9), and as such analyses in this paper focus on two pollutants for statistical parsimony and to minimise multiple testing: PM_2.5_ and NO_2_, which are known to cause adverse health effects and have been previously evaluated for association with mental health outcomes [43].

The following sequence of models was fitted to test the association between each ambient air pollutant and trajectories of conduct problems in adolescence, and the moderation of the association by gender, racism, and ethnicity. Model 1 contained each ambient air pollutant at the intercept (centred at age 12), age as a fixed effect and an interaction between each ambient air pollutant and the slope for age; Model 2 adjusted for all covariables to explore whether any association between each ambient air pollutant and conduct problems at age 12, as well as an association between ambient air pollution and the rate of change in conduct problems during adolescence was explained by their adjustment. Models 3 and 4 adjusted for interactions between each air pollutant, racism, and ethnicity. Model 5 considered the simultaneous interactions between each air pollutant and ethnicity. Statistical interactions were assessed using Wald tests. Models 2 and 5 are shown, since they are key models.

Multiple imputation by chained equations was used to deal with missing data in all analyses under the missing at random assumption. Sixty imputed datasets were created using regression switching. The imputation model included all exposures (residential air pollutants), effect modifiers (racism and ethnicity), covariables (sex, age in years at survey time, parenting style, maternal mental health, adolescent health behaviours, family affluence, family type, and labour market status neighbourhood deprivation), the outcome (conduct problems), and auxiliary variables (generational status and hyperactivity problems) to help predict missing data. Imputation models also included interaction terms between residential air pollutants, racism and ethnicity. The seed number was set at 03052020. Results were combined using Rubin’s rules [44]. Results from imputed data are reported.

## Results

### Descriptive results

Table 1 shows that Black Caribbean and Black African adolescents were exposed to higher levels of PM_2.5_ than their White British peers at baseline survey, while Black African adolescents were exposed to higher PM_2.5_ concentrations at follow-up. Black Caribbean and Black African adolescents were also exposed to higher NO_2_ concentrations than their White British peers at both baseline and follow-up surveys. Black Caribbean adolescents had higher conduct problems, whereas Indian adolescents had lower conduct problems than their White British counterparts. All ethnic minority groups reported more racism than White British adolescents (which increased with age). Ethnic minority adolescents were also more likely to live in materially less-advantaged households, and to live in more deprived neighbourhoods than White British participants.

**Table 1:** Descriptive profile of the DASH participants by ethnicity^§^

Variable	DASH 11–13y							DASH 14–16y						
	All (*n* = 4775)	White British (*n* = 872)	Black Caribbean (*n* = 713)	Black African (*n* = 842)	Indian (*n* = 397)	Pakistani& Bangladeshi (*n* = 460)	Others (*n* = 1491)	All (*n* = 4775)	White British (*n* = 872)	Black Caribbean (*n* = 713)	Black African (*n* = 842)	Indian (*n* = 397)	Pakistani & Bangladeshi (n = 460)	Others (n = 1491)
	Mean (95% CI)	Mean (95% CI)	Mean (95% CI)	Mean (95% CI)	Mean (95% CI)	Mean (95% CI)	Mean (95% CI)	Mean (95% CI)	Mean (95% CI)	Mean (95% CI)	Mean (95% CI)	Mean (95% CI)	Mean (95% CI)	Mean (95% CI)
Conduct problems score	2.4 (2.4 to 2.5)	2.5 (2.3 to 2.6)	2.7 (2.6 to 2.8)	2.4 (2.3 to 2.5)	2.1 (2.0 to 2.3)	2.2 (2.0 to 2.3)	2.5 (2.4 to 2.5)	2.4 (2.3 to 2.4)	2.3 (2.2 to 2.5)	2.5 (2.4 to 2.7)	2.4 (2.3 to 2.5)	2.0 (1.9 ro 2.2)	2.2 (2.1 to 2.4)	2.4 (2.3 to 2.5)
Air pollutants														
PM_2.5_	19.4 (19.4 to 19.4)	19.2 (19.2 to 19.2)	19.5 (19.5 to 19.5)	19.6 (19.6 to 19.6)	19.3 (19.3 to 19.3)	19.4 (19.4 to 19.4)	19.5 (19.5 to 19.5)	16.1 (16.1 to 16.1)	15.9 (15.9 to 15.9)	16.1 (16.1 to 16.1)	16.2 (16/2 to 16.3)	15.9 (15.9 to 15.9)	16.0 (16.0 to 16.1)	16.1 (16.1 to 16.1)
NO_2_	41.6 (41.5 to 41.7)	40 .0(39.7 to 40.3)	42. 0(41.7 to 42.3)	42.9 (42.6 to 43.1)	40.5 (40.2 to 40.8)	41.0 (40.7 to 41.3)	42. 1 (41.9 to 42.3)	40.8 ( 40.7 to 40.9)	39.3 (39.0 to 39.6)	41.1 (40.8 to 41.4)	42. 0 (41.6 to 42.3)	39.5 (39.2 to 39.9)	40.5 (40.1 to 40.8)	41.3 (41.0 to 41.5)
	% (95% CI)	% (95% CI)	% (95% CI)	% (95% CI)	% (95% CI)	% (95% CI)	% (95% CI)	% (95% CI)	% (95% CI)	% (95% CI)	% (95% CI)	% (95% CI)	% (95% CI)	% (95% CI)
Racism	28.2 (24.9 to 31.5)	15.3 (12.7 to 17.8)	22.5 (18.9 to 26.2)	27.1 (23.6 to 30.6)	25.3 (20.5 to 30.0)	32.9 (28.1 to 37.6)	38.3 (29.6 to 46.9)	34.7 (33.0 to 35.7)	24.0 (21.2 to 26.8)	37.0 (33.3 to 40.6)	40.1 (36.7 to 43.4)	37.9 (33.0 42.6)	36.2 (31.2 to 40.6)	34.6 (32.1 to 37.0)
Socioeconomic circumstances														
Family Affluence Scale														
Least advantaged	17.0 (15.9 to 18.2)	13.7 (11.3 to 16.1)	21.2 (17.9 to 24.5)	17.3 (14.5 to 20.0)	10.7 (7.3 to 14.1)	14.1 (10.7 to 17.5)	19.5 (17.3 to 21.6)	11.9 (11.0 to 12.9)	10.8 (8.7 to 12.9)	17.8 (15.0 to 20.7)	12.0 (9.8 to 14.3)	6.3 (3.8 to 8.7)	7.6 (5.1 to 10.0)	12.6 (10.8 to 14.3)
Least disadvantaged	61.5 (60.0 to 62.9)	68.7 (65.4 to 71.8)	56.1 (52.2 to 60.0)	62.8 (59.3 to 66.3)	64.9 (59.8 to 70.0)	60.1 (55.3 to 64.5)	58.5 (55.8 to 61.1)	68.3 (66.9 to 69.6)	73.4 (70.5 to 76.4)	62.3 (58.7 to 65.9)	68.5 (65.2 to 71.6)	74.9 (70.6 to 79.3)	71.5 (67.3 to 75.7)	65.3 (62.8 to 67.8)
IMD-income domain (quintiles)														
Least deprived quintile	26.3 (25.1 to 27.6)	43.8 (40.4 to 47.1)	26.4 (23.2 to 47.1)	13.9 (11.6 to 16.3)	35.0 (30.3 to 39.7)	20.0 (16.4 to 23.7)	22.7 (20.6 to 24.9)	18.7 (17.8 to 20.0)	37.2 (33.9 to 40.4)	15.3 (12.6 to 17.9)	11.2 (9.1 to 13.4)	19.8 (15.9 to 23.8)	11.3 (8.4 to 14.2)	16.3 (14.4 to 18.1)
Most deprived quintile	13.3 (12.3 to 14.3)	8.5 (6.7 to 10.4)	14.3 (11.7 to 16.8)	20.3 (17.6 to 23.1)	4.1 (2.1 to 6.0)	7.2 (4.9 to 9.6)	16.1 (14.2 to 17.9)	21.4 (20.3 to 22.6)	13.7 (11.4 to 16.0)	22.5 (19.4 to 25.5)	28.3 (25.2 to 31.3)	12.1 (8.9 to 15.4)	16.0 (13.0 to 19.8)	25.6 (23.3 to 27.8)

Descriptive characteristics of all the variables used in the statistical analysis are shown in Supplementary Table 1

### Ambient air pollution and trajectories of adolescent conduct problems

Over time, higher PM_2.5_ and NO_2_ concentrations were associated with a flattened trajectory of conduct problems, while lower of PM_2.5_ and NO_2_ concentrations were associated with a decrease in conduct problems over time (Fig. 1). Table 2 shows that PM_2.5_ was associated with a greater rate of change of conduct problems during adolescence was (*β* = 0.06 (95% CI 0.03 to 0.08) *p* < 0.001) than NO_2_ (*β* = 0.01 (95% CI 0.00 to 0.01) *p* < 0.001), suggesting a stronger link between PM_2.5_ and change in conduct problems during adolescence. Adjustment for demographic characteristics, racism, psychosocial factors, health behaviours, maternal mental health problems, parenting style, individual, and area-level characteristics did not explain these associations.

**Table 2:** Pooled and interactive effects of air pollution on trajectories of adolescent conduct problems

	PM 2.5 and conduct problems		NO_2_ and conduct problems	
	Model 2	Model 5	Model 2	Model 5
	Coefficient (95% confidence interval)	Coefficient (95% confidence interval)	Coefficient (95% confidence interval)	Coefficient (95% confidence interval)
Fixed effects				
Pollutant	− 0.11 (− 0.17 to − 0.05)***	− 0.09 (− 0.16 to − 0.02)**	− 0.02 (− 0.03 to 0.00)*	− 0.03 (− 0.05 to 0.01)*
Age	− 1.22 (− 1.60 to − 0.84)***	− 1.16 (− 1.55 to − 0.78)	− 0.48 (− 0.69 to − 0.27)***	− 0.48 (− 0.69 to − 0.27)***
Pollutant*age	0.06 (0.03 to 0.08)***	0.06 (0.04 to 0.08)***	0.01 (0.00 to 0.01)***	0.01 (0.00 to 0.01)**
Female	− 0.36 (− 0.43 to − 0.28) ***	− 0.92 (− 1.47 to − 0.25)**	− 0.36 (− 0.43 to − 0.28)***	− 0.36 (− 0.43 to − 0.28)***
Racism	0.36 (0.28 to 0.44)***	− 0.61 (− 1.33 to 0.10)	0.36 (0.28 to 0.43)***	0.44 (− 0.30 to 1.18)
Ethnicity (Ref. White British)				
Black Caribbean	0.20 (0.07 to 0.33)**	0.65 (− 0.31 to 1.60)	0.22 (0.09 to 0.35)**	0.00 (− 1.22 to 1.24)
Black African	0.12 (− 0.01 to 0.25)	1.34 (0.41 to 2.26)**	0.13 (0.00 to 0.26)	− 0.57 (− 1.71 to 0.56)
Indian	− 0.13 (− 0.29 to 0.02)	0.59 (− 0.51 to 1.71)	− 0.15 (− 0.31 to 0.01)	− 0.23 (− 1.94 to 1.48)
Pakistani & Bangladeshi	− 0.05 (− 0.20 to 0.10)	1.81 (0.71 to 2.92)**	− 0.08 (− 0.23 to 0.07)	0.47 (− 1.17 to 2.12)
Others	0.04 (− 0.07 to 0.15)	1.24 (0.41 to 2.08)**	0.04 (− 0.07 to 0.15)	− 0.24 (− 1.27 to 0.79)
Moderation				
Pollutant*racism		0.05 (0.01 to 0.10)**		0.00 (− 0.02 to 0.02)
Pollutant*ethnicity (Ref. White British)				
Black Caribbean		− 0.02 (− 0.08 to 0.03)		0.00 (− 0.02 to 0.03)
Black African		− 0.07 (− 0.12 to − 0.02)**		0.02 (− 0.025 to 0.03)
Indian		− 0.04 (− 0.10 to 0.02)		0.00 (− 0.04 to 0.04)
Pakistani & Bangladeshi		− 0.11 (− 0.17 to − 0.04)**		− 0.01 (− 0.05 to 0.03)
Others		− 0.07 (− 0.12 to − 0.02)**		0.01 (− 0.02 to 0.032)
Random effects				
Level 2 (Between-child intercept variance)	0.89 (0.85 to 0.94)	0.89 (0.85 to 0.93)	0.89 (0.85 to 0.93)	0.89 (0.85 to 0.93)
Level 1 (occasion)	1.25 (1.22 to 1.28)	1.25 (1.22 to 1.29)	1.26 (1.23 to 1.28)	1.26 (1.23 to 1.28)

All statistical models are shown in supplementary Tables 2 and 3

^*^ p < 0.05, **p < 0.01, *** p < 0.001

Model 2—Ambient air pollutant, age, ambient air pollutant * age, racism, sex, ethnicity, maternal mental health problems, parental care, parental control, cigarette smoking, alcohol, family affluence and IMD (income domain)

Model 5—Model 2 + ambient air pollutant *racism + ambient air pollutant * ethnicity

### Effect modification by racism and ethnicity

**Fig. 2:**
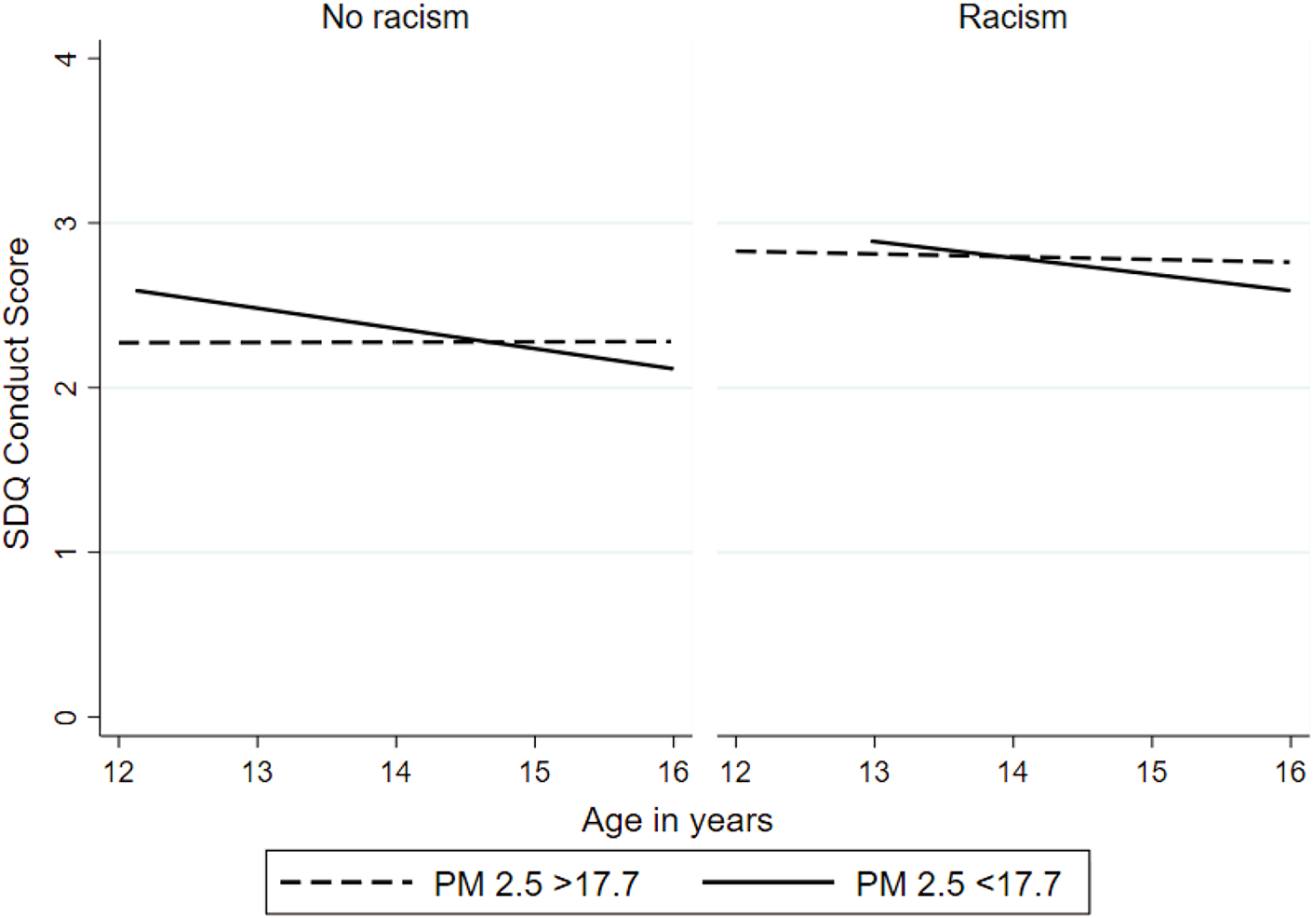
Predicted conduct problem trajectories by self-reported racism and PM_2.5_ concentration level (Model 5). Predictions are plotted as below average (PM_2.5_ < 17.7 mg/m^3^) and above average (PM_2.5_ > 17.7 mg/m^3^) for DASH participants who experienced racism and those who were not. Predictions were restricted between ages 12 and 16 years where measures of PM_2.5_ and conduct scores were more robust

**Fig. 3:**
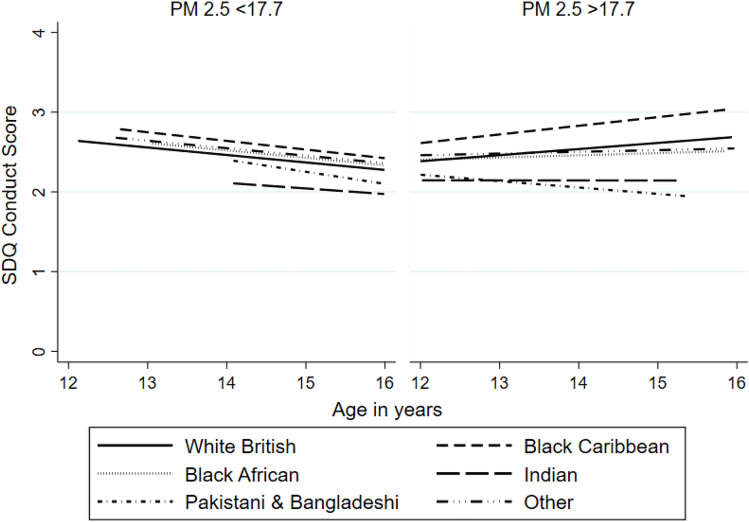
Predicted conduct problem trajectories by ethnicity and PM_2.5_ concentration level (Model 5). Predictions are plotted as below average (PM_2.5_ < 17.7 mg/m^3^) and above average (PM_2.5_ > 17.7 mg/m^3^) PM_2.5_ concentration level for each ethnic group. Predictions were restricted between ages 12 and 16 years where measures of PM_2.5_ and conduct scores were more robust

Model 3 showed that racism was associated with an amplification of the relationship betweenPM_2.5_ and adolescent trajectories of conduct problems over time (see Fig. 2), while Model 4 indicated a divergence of trajectories of adolescent conduct problems associated to higher levels of PM_2.5_ between ethnic minority groups (see Fig. 3), with White British and Black Caribbean adolescents experiencing an increase in conduct problems over time.

Adjustment for demographic characteristics, psychosocial factors, health behaviours, individual- and area-level characteristics, as well as mutual adjustment for statistical interactions with racism, and ethnicity (Model 5) did not affect the interpretation of the findings. No significant interactions were observed for NO_2_ (see Table 2 and Supplementary Table 3)_._

### Sensitivity analysis

Analyses of non-imputed data did not affect the pattern and the significance of findings shown in Model 2 (association between PM_2.5_ and conduct problems). The pattern of findings was maintained for the interactions between PM_2.5_, racism, and ethnicity (Model 5), but significance was lost (see Supplementary Table 4).

Further sensitivity analyses explored whether there was evidence of a modifying of sex on the interplay between PM_2.5_ or NO_2_, racism, and ethnicity on adolescent trajectories of conduct problems. No evidence was provided for effect modification.

We repeated analyses using a two-pollutant model (PM_2.5_ and NO_2_) to examine co-pollutant confounding in Model 5 (see Supplementary Table 5). The associations arising from PM_2.5_ remained relatively unchanged when simultaneously modelled with NO_2_, while the associations arising from NO_2_ were null when simultaneously modelled with PM_2.5_. This finding suggests that associations arising from PM_2.5_ were not driven by NO_2_ or another factor correlated with NO_2,_ while associations arising from NO_2_ were confounded by PM_2.5_.

Finally, we considered potential associations between racism, exposure to higher PM_2.5_ concentrations, and adolescents’ attention deficit/hyperactivity problems. No interaction was observed (results not shown), highlighting the possible exacerbating role of racism only on adolescents’ conduct problems who are exposed to higher PM_2.5_ concentrations.

## Discussion

### Statement of principal findings

Using data from one of the largest known and most diverse longitudinal multi-ethnic cohort studies of adolescents in the UK, we found that exposure to higher PM_2.5_ and NO_2_ concentrations was associated with a flattened trajectory of conduct problems, while exposure to lower PM_2.5_ and NO_2_ concentrations was associated with a decrease in conduct problems. Among adolescents who were exposed to higher PM_2.5_ concentrations_,_ racism was associated with an increase in conduct problems, but not with attention deficit/hyperactivity problems. Finally, exposure to higher PM_2.5_ concentrations was associated with an exacerbation of ethnic differences in conduct problems during adolescence for White and Black Caribbean participants.

### Strengths and weaknesses of the study

DASH has high retention rates due to enormous community support. The cohort’s composition is unique with large numbers from the main UK ethnic groups. Weaknesses include the lack of data before age 11 years, the lack of information on individual-level exposure to ambient air pollution, potential residual environmental confounding such as lack of exposure to green space or data to inform the mechanisms linking air pollution and conduct problems in adolescence such as noise pollution, sleep or inflammatory biomarkers, and lack of statistical power to explore three-way interactions between time, air pollutants, and racism or ethnicity.

### Interpretation

The association between long-term PM_2.5_ exposures with conduct problems aligns with previous evidence from animal studies, where phenotypes associated with impulsivity (a behaviour characterised by little or no reflection, or consideration of the consequences) were more prevalent in mice with early life exposures to concentrated ambient ultrafine particles [45]. Although mechanisms of these effects are not fully elucidated, such neurobehavioral effects may be mediated by particle-induced alteration of the mesocorticolimbic dopamine system associated with impulsive-antisocial behaviour [45].

There is a difficulty in comparing this study’s findings with those from other human studies. First, there is a paucity of follow-up studies exploring the association between air pollutants and mental health in adolescents. Second, a few studies exist with a focus on air pollution and conduct problems. Third, different scales and criteria have been used to measure conduct problems in adolescence.

The association between PM_2.5_ and higher conduct problems in adolescence corroborates the findings of a US longitudinal study in South California following 682 participants from age 9 to age 18 years [20]. This study reported that interquartile increases in PM_2.5_ by 3.12–5.18 μg/m^3^ were associated with higher conduct problems at age 9 years using the Child Behaviour Checklist (CBCL), but not with the rate of change of conduct problems. A cross-sectional study of 174 participants aged 7–14 years in Boston, US found that that traffic-related black carbon was associated with increased impulsivity using the Conner’s Continuous Performance Test, with the effects being greater for boys [46]. Conversely, a cross-sectional study of 284 participants in London reported no association between higher concentrations of PM_2.5_ and NO_2_ at age 12 and higher conduct problems at age 12 and 6 years later at age 18 using DSM-IV criteria [17]. Finally, a meta-analysis of 13,182 participants aged 7–11 years in five European countries (The Netherlands, Germany, Poland, France, and Spain) and from 8 birth cohort studies did not find evidence of associations between exposure to prenatal and postnatal increases in NO_2_ and PM_2.5_ concentrations with a greater likelihood of probable clinical conduct problems using the CBCL and the SDQ [47].

Our study provides novel insights into the development of adolescent conduct problems as well as ethnic differences in the development of conduct problems in response to exposure to PM_2.5_ concentrations. They also further our understanding of the synergies between PM_2.5_ and racism in the development conduct problems during the sensitive period of adolescence [48]. Some argue that an emotional mechanism, in which anger and belief in the legitimacy of violence act as indirect links, connects racism and conduct problems [49]. Since racism may elicit these attitudes, adolescents may become more likely to affiliate with peers who have similar outlooks. Therefore, social selection effects such as these may lead to social influence effects, which may further influence conduct problems in adolescence [50, 51]. There is also increasing evidence highlighting that psychological stress may enhance a child’s vulnerability to certain chemical exposures, signalling the importance of studying interactions of social-chemical stressors [52]. In our study, the PM_2.5_-conduct problems’ association was stronger in adolescents who reported racism. However, the neurobiological mechanisms underlying the amplification of adverse neurobehavioral effects of airborne particles by self-reported racism are unclear. It is possible that adolescents who endure ongoing racism are in a prolonged state of toxic stress [53]. Growing evidence suggests that chronic stress may act upon one or more of the same critical physiological pathways as air pollutants, including oxidative stress, inflammation, and autonomic disruption [52].

## Conclusions

Our study contributes new evidence on the association between PM_2.5_ and conduct problems during the sensitive period of adolescence, and on the interplay between ambient PM_2.5_, racism, and ethnicity in the development of adolescent conduct problems.

## Data sharing statement

The DASH data are available to researchers via a data request to the MRC Social and Public Health Science Unit. Applications and the data sharing policy for DASH can be found at http://dash.sphsu.mrc.ac.uk/DASH_dsp_v1_November-2012_draft.pdf. It reflects the MRC guidance on data sharing with the aim of making the data as widely and freely available as possible while safeguarding the privacy of participants, protecting confidential data, and maintaining the reputation of the study. All potential collaborators work with a link person, an experienced DASH researcher—to support their access to and analysis of the data. The variable-level metadata is available from the study team and via the MRC Data Gateway.

## Supplementary Information

Below is the link to the electronic supplementary material.

Supplementary file 1 (DOCX 4961 KB)
