# Supplementary material for: Air pollution and trajectories of adolescent conduct problems: the roles of ethnicity and racism; evidence from the DASH longitudinal study
Source: Soc Psychiatry Psychiatr Epidemiol. 2021 Apr 30;56(11):2029–39. doi: 10.1007/s00127-021-02097-7 (PMC8519907; doi:10.1007/s00127-021-02097-7)
Supplement: Supplementary file 1 — (DOCX 4961 KB) [file 127_2021_2097_MOESM1_ESM.docx]

***Supplementary Table 1:*** *Descriptive profile of the DASH participants by ethnicity*

|  |  | **DASH 11-13y** | | | | | |  | **DASH 14-16y** | | | | | |
| --- | --- | --- | --- | --- | --- | --- | --- | --- | --- | --- | --- | --- | --- | --- |
| **Variable** | **All (n=4775)** | **White British (n=872)** | **Black Caribbean (n=713)** | **Black African (n=842)** | **Indian (n=397)** | **Pakistani& Bangladeshi (n=460)** | **Others (n=1491)** | **All (n=4775)** | **White British (n=872)** | **Black Caribbean (n=713)** | **Black African (n=842)** | **Indian (n=397)** | **Pakistani & Bangladeshi (n=460)** | **Others (n=1491)** |
|  | Mean (95% CI) | Mean (95% CI) | Mean (95% CI) | Mean (95% CI) | Mean (95% CI) | Mean (95% CI) | Mean (95% CI) | Mean (95% CI) | Mean (95% CI) | Mean (95% CI) | Mean (95% CI) | Mean (95% CI) | Mean (95% CI) | Mean (95% CI) |
| **Conduct score** | 2.4 (2.4 to 2.5) | 2.5 (2.3 to 2.6) | 2.7 (2.6 to 2.8) | 2.4 (2.3 to 2.5) | 2.1 (2.0 to 2.3) | 2.2 (2.0 to 2.3) | 2.5 (2.4 to 2.5) | 2.4 (2.3 to 2.4) | 2.3 (2.2 to 2.5) | 2.5 (2.4 to 2.7) | 2.4 (2.3 to 2.5) | 2.0 (1.9 ro 2.2) | 2.2 (2.1 to 2.4) | 2.4 (2.3 to 2.5) |
| **Air pollutants** | | | | | | | | | | | | | | |
| PM_2.5_ | 19.4 (19.4 to 19.4) | 19.2 (19.2 to 19.2) | 19.5 (19.5 to 19.5) | 19.6 (19.6 to 19.6) | 19.3 (19.3 to 19.3) | 19.4 (19.4 to 19.4) | 19.5 (19.5 to 19.5) | 16.1 (16.1 to 16.1) | 15.9 (15.9 to 15.9) | 16.1 (16.1 to 16.1) | 16.2 (16/2 to 16.3) | 15.9 (15.9 to 15.9) | 16.0 (16.0 to 16.1) | 16.1 (16.1 to 16.1) |
| NO_2_ | 41.6 (41.5 to 41.7) | 40 .0(39.7 to 40.3) | 42. 0(41.7 to 42.3) | 42.9 (42.6 to 43.1) | 40.5 (40.2 to 40.8) | 41.0 (40.7 to 41.3) | 42. 1 (41.9 to 42.3) | 40.8 ( 40.7 to 40.9) | 39.3 (39.0 to 39.6) | 41.1 (40.8 to 41.4) | 42. 0 (41.6 to 42.3) | 39.5 (39.2 to 39.9) | 40.5 (40.1 to 40.8) | 41.3 (41.0 to 41.5) |
|  | % (95% CI) | % (95% CI) | % (95% CI) | % (95% CI) | % (95% CI) | % (95% CI) | % (95% CI) | % (95% CI) | % (95% CI) | % (95% CI) | % (95% CI) | % (95% CI) | % (95% CI) | % (95% CI) |
| **Racism** | 28.2 (24.9 to 31.5) | 15.3 (12.7 to 17.8) | 22.5 (18.9 to 26.2) | 27.1 (23.6 to 30.6) | 25.3 (20.5 to 30.0) | 32.9 (28.1 to 37.6) | 38.3 (29.6 to 46.9) | 34.7 (33.0 to 35.7) | 24.0 (21.2 to 26.8) | 37.0 (33.3 to 40.6) | 40.1 (36.7 to 43.4) | 37.9 (33.0 42.6) | 36.2 (31.2 to 40.6) | 34.6 (32.1 to 37.0) |
| **Maternal mental health problems** | 5.4 (4.7 to 6.2) | 5.5 (3.4 to 7.2) | 5.5 (4.0 to 8.0) | 3.0 (1.8 to 4.4) | 4.2 (2.0 to 6.3) | 3.3 (1.4 to 5.2) | 7.5 (5.9 to 9.0) | 6.3 (5.6 to 7.1) | 7.7 (5.8 to 9.6) | 3.9 (2.3 to 5.4) | 4.1 (2.6 to 5.5) | 7.1 (4.4 to 9.7) | 5.0 (2.9 to 7.1) | 8.2 (6.7 to 9.7) |
| **Parental care** | | | | | | | | | | | | | | |
| High | 40.2 (38.7 to 41.6) | 41.8 (38.5 to 45.2) | 41.3 (37.5 to 45.1) | 37.2 (33.8 to 40.5) | 41.3 (36.4 to 46.3) | 40.3 (35.8 to 44.9) | 40.2 (37.6 to 42.8) | 23.6 (22.4 to 24.8) | 25.4 (22.5 to 28.3) | 19.7 (16.7 to 22.6) | 19.8 (17.1 to 22.6) | 27.9 (23.5 to 32.3) | 28.6 (24.4 to 32.7) | 23.8 (21.6 to 25.9) |
| Medium | 29.4 (28.1 to 30.7) | 33.4 (30.2 to 36.6) | 26.0 (22.6 to 29.4) | 28.0 (24.8 to 31.1) | 27.8 (23.3 to 32.2) | 29.5 (25.3 to 33.8) | 30.0 (27.4 to 32.2) | 27.7 (26.4 to 28.9) | 29.2 (26.1 to 32.2) | 27.6 (24.3 to 30.9) | 26.0 (23.0 to 29.0) | 30.2 (25.6 to 34.7) | 26.3 (22.2 to 30.4) | 27.5 (25.2 to 29.8) |
| **Low** | 30.4 (29.0 to 31.7) | 24.8 (21.9 to 27.7) | 32.7 (29.1 to 36.2) | 34.8 (31.5 to 38.2) | 30.8 (26.2 to 35.5) | 30.2 (25.9 to 34.8) | 29.9 (27.5 to 32.2) | 48.8 (47.3 to 50.2) | 45.4 (42.1 to 48.8) | 52.8 (49.1 to 56.6) | 54.2 (50.7 to 57.6) | 41.9 (37.1 to 46.8) | 45.2 (40.6 to 49.8) | 48.7 (46.1 to 51.2) |
| **Parental control** | | | | | | | | | | | | | | |
| Low | 27.1 (25.8 to 28.4) | 39.5 (36.2 to 42.8) | 29.1 (25.6 to 32.6) | 20.8 (18.0 to 23.6) | 23.7 (19.4 to 27.9) | 19.4 (15.6 to 23.0) | 25.9 (23.5 to 28.2) | 30.3 (29.0 to 31.6) | 44.5 (41.2 to 47.8) | 29.6 (26.2 to 32.9) | 23.8 (20.9 to 26.8) | 26.1 (21.8 to 30.4) | 22.4 (18.5 to 26.2) | 29.6 (27.3 to 31.9) |
| Medium | 38.5 (37.1 to 39.9) | 39.3 (36.5 to 39.3) | 36.2 (32.5 to 39.9) | 37.0 (33.7 to 40.4) | 38.0 (33.2 to 42.4) | 38.9 (34.3 to 43.3) | 39.7 (37.2 to 42.3) | 33.9 (32.6 to 35.3) | 32.8 (29.6 to 35.9) | 33.4 (30.0 to 36.9) | 33.1 (30.0 to 36.4) | 33.9 (29.3 to 38.6) | 36.4 (32.0 to 40.9) | 34.5 (32.0 to 37.0) |
| High | 34.4 (33.0 to 33.8) | 20.6 (17.9 to 23.4) | 34.8 (31.1 to 38.4) | 42.1 (38.7 to 45.6) | 38.4 (33.5 to 43.1) | 41.7 (37.2 to 46.4) | 34.3 (31.9 to 37.0) | 35.7 (34.4 to 37.1) | 22.6 (19.9 to 25.5) | 37. 0 (33.4 to 40.6) | 43.0 (39.6 to 46.4) | 39.9 (35.1 to 44.8) | 41.2 (36.7 to 45.7) | 35.9 (33.4 to 38.3) |
| **Smoking** | 7.5 (6.5 to 8.4) | 11.1 (8.7 to 13.5) | 10.1 (7.1 to 12.8) | 2.0 (0.7 to 3.2) | 1.9 (0.1 to 3.7) | 5.4 (2.2 to 8.0) | 8.5 (6.7 to 10.4) | 17.7 (16.4 to 19.0) | 32.2 (28.7 to 35.6) | 16.1 (12.9 to 19.3) | 7.9 (4.7 to 10.0) | 7.9 (4.7 to 10.9 | 14.1 (10.5 to 17.6) | 18.7 (14.5 to 20.9) |
| **Alcohol** | 31.0 (29.6 to 32.5) | 53.1 (49.7 to 56.6) | 45.5 (41.4 to 49.4) | 20.4 (17.4 to 23.4) | 9.0 (6.0 to 12.0) | 1.9 (0.1 to 3.1) | 32 (29.4 to 34.5) | 50.3 (49.0 to 51.7) | 78.8 (76.0 to 81.5) | 65.1 (61.6 to 68.7) | 35.2 (31.9 to 38.4) | 33.4 (28.7 to 38.0) | 5.1 (3.0 to 7.1) | 53.6 (51.1 to 56.2) |
| **Socioeconomic circumstances** | | | | | | | | | | | | | | |
| **Family Affluence Scale** | | | | | | | | | | | | | | |
| Least advantaged | 17.0 (15.9 to 18.2) | 13.7 (11.3 to 16.1) | 21.2 (17.9 to 24.5) | 17.3 (14.5 to 20.0) | 10.7 (7.3 to 14.1) | 14.1 (10.7 to 17.5) | 19.5 (17.3 to 21.6) | 11.9 (11.0 to 12.9) | 10.8 (8.7 to 12.9) | 17.8 (15.0 to 20.7) | 12.0 (9.8 to 14.3) | 6.3 (3.8 to to 8.7) | 7.6 (5.1 to 10.0) | 12.6 (10.8 to 14.3) |
| Less advantaged | 21.5 (20.3 to 22.8) | 17.6 (15.0 to 20.3) | 22.7 (19.4 to 26.0) | 19.9 (17.0 to 22.9) | 24.4 (19.9 to 29.0) | 25.8 (21.5 to 30.0) | 22.0 (19.7 to 24.3) | 19.8 (18.6 to 20.9) | 15.8 (13.3 to 18.2) | 19.8 (16.8 to 22.8) | 19.6 (16.8 to 22.3) | 18.7 (14.8 to 22.6) | 20.9 (17.2 to 24.7) | 22.1 (20.0 to 24.3) |
| Least disadvantaged | 61.5 (60.0 to 62.9) | 68.7 (65.4 to 71.8) | 56.1 (52.2 to 60.0) | 62.8 (59.3 to 66.3) | 64.9 (59.8 to 70.0) | 60.1 (55.3 to 64.5) | 58.5 (55.8 to 61.1) | 68.3 (66.9 to 69.6) | 73.4 (70.5 to 76.4) | 62.3 (58.7 to 65.9) | 68.5 (65.2 to 71.6) | 74.9 (70.6 to 79.3) | 71.5 (67.3 to 75.7) | 65.3 (62.8 to 67.8) |
| **IMD-Income domain (quintiles)** | | | | | | | | | | | | | | |
| Least deprived quintile | 26.3 (25.1 to 27.6) | 43.8 (40.4 to 47.1) | 26.4 (23.2 to 47.1) | 13.9 (11.6 to 16.3) | 35.0 (30.3 to 39.7) | 20.0 (16.4 to 23.7) | 22.7 (20.6 to 24.9) | 18.7 (17.8 to 20.0) | 37.2 (33.9 to 40.4) | 15.3 (12.6 to 17.9) | 11.2 (9.1 to 13.4) | 19.8 (15.9 to 23.8) | 11.3 (8.4 to 14.2) | 16.3 (14.4 to 18.1) |
| 2nd | 20.2 (19.0 to 21.3) | 20.2 (17.5 to 22.9) | 19.8 (16.8 to 22.7) | 13.8 (11.5 to 16.2) | 26.5 (22.2 to 30.9) | 26.6 (22.2 to 30.9) | 20.2 (18.2 to 22.2) | 18.6 (17.5 to 20.0) | 17.2 (14.7 to 19.7) | 22.5 (19.5 to 25.6) | 14.9 (12.5 to 17.3) | 28.3 (23.9 to 32.8) | 18.9 (15.4 to 22.5) | 16.8 (14.9 to 18.7) |
| 3rd | 20.5 (19.4 to 21.7) | 15.3 (12.9 to 17.8) | 18.8 (15.9 to 21.7) | 23.4 (20.5 to 26.3) | 22.2 (18.1 to 26.3) | 19.2 (17.1 to 21.1) | 12.2 (10.0 to 14.4) | 19.2 (18.1 to 20.3) | 18.0 (15.4 to 20.5) | 20.5 (17.5 to 23.5) | 18.3 (15.6 to 20.9) | 19.7 (15.8 to 23.6) | 23.5 (19.6 to 27.4) | 18.3 (16.3 to 20.3) |
| 4th | 19.7 (18.5 to 20.8) | 12.2 (10.0 to 14.4) | 20.8 (17.8 to 23.8) | 28.5 (25.5 to 31.6) | 12.1 (8.9 to 15.4) | 15.5 (12.2 to 18.8) | 21.8 (19.7 to 23.9) | 22.0 (20.8 to 23.2) | 14.0 (11.7 to 16.3) | 19.2 (16.3 to 22.1) | 27.4 (24.3 to 30.4) | 20.0 (16.0 to 23.9) | 29.8 (25.6 to 34.0) | 23.0 (20.9 to 25.2) |
| Most deprived quintile | 13.3 (12.3 to 14.3) | 8.5 (6.7 to 10.4) | 14.3 (11.7 to 16.8) | 20.3 (17.6 to 23.1) | 4.1 (2.1 to 6.0) | 7.2 (4.9 to 9.6) | 16.1 (14.2 to 17.9) | 21.4 (20.3 to 22.6) | 13.7 (11.4 to 16.0) | 22.5 (19.4 to 25.5) | 28.3 (25.2 to 31.3) | 12.1 (8.9 to 15.4) | 16.0 (13.0 to 19.8) | 25.6 (23.3 to 27.8) |

***Supplementary Table 2:*** *Pooled and interactive effects of PM 2.5 on trajectories of adolescent conduct problems.*

|  | Model 1 | Model 2 | Model 3 | Model 4 | Model 5 |
| --- | --- | --- | --- | --- | --- |
|  | **Coefficient (95% confidence interval)** | **Coefficient (95% confidence interval)** | **Coefficient (95% confidence interval)** | **Coefficient (95% confidence interval)** | **Coefficient (95% confidence interval)** |
| Fixed effects | | | | | |
| **Pollutant** | -0.07 (-0.13 to -0.01)* | -0.11 (-0.17 to -0.05)*** | -0.13 (-0.19 to -0.07)*** | -0.07 (-0.13 to 0.00) | -0.09 (-0.16 to -0.02) |
| **Age** | -0.87 (-1.25 to -0.49)*** | -1.22 (-1.60 to -0.84)*** | -1.21 (-1.60 to -0.82)*** | -1.19 (-1.57 to -0.80)*** | -1.16 (-1.55 to -0.78) |
| **Pollutant*age** | 0.05 (0.03 to 0.07)*** | 0.06 (0.03 to 0.08)*** | 0.06 (0.04 to 0.08)*** | 0.06 (0.04 to 0.08)*** | 0.06 (0.04 to 0.08)*** |
| **Racism** |  | 0.36 (0.28 to 0.44)*** | -0.41 (-1.12 to 0.30) | 0.36 (0.28 to 0.44)*** | -0.61 (-1.33 to 0.10) |
| **Female** |  | -0.36 (-0.43 to -0.28)*** | -0.35 (-0.43 to -0.28)*** | -0.36 (-0.43 to -0.28)*** | -0.36 (-0.43 to -0.28)*** |
| **Ethnicity (Ref. White British)** | | | | | |
| Black Caribbean |  | 0.20 (0.07 to 0.33)** | 0.19 (0.07 to 0.33)** | 0.49 (-0.46 to 1.45) | 0.65 (-0.31 to 1.60) |
| Black African |  | 0.12 (-0.01 to 0.25) | 0.12 (-0.01 to 0.25) | 1.13 (0.22 to 2.05)* | 1.34 (0.41 to 2.26)** |
| Indian |  | -0.13 (-0.29 to 0.02) | -0.14 (-0.30 to 0.02) | 0.47 (-0.64 to 1.58) | 0.59 (-0.51 to 1.71) |
| Pakistani & Bangladeshi |  | -0.05 (-0.20 to 0.10) | -0.06 (-0.21 to 0.09) | 1.72 (0.63 to 2.81)** | 1.81 (0.71 to 2.92)** |
| Others |  | 0.04 (-0.07 to 0.15) | 0.03 (-0.08 to 0.15) | 1.06 (0.24 to 1.88) | 1.24 (0.41 to 2.08)** |
| **Maternal mental health problems** |  | 0.23 (0.07 to 0.39)** | 0.23 (0.07 to 0.38)** | 0.23 (0.07 to 0.38)** | 0.23 (0.07 to 0.38)** |
| **Parental care (Ref. High)** | | | | | |
| Medium |  | 0.26 (0.18 to 0.34)*** | 0.26 (0.18 to 0.34)*** | 0.26 (0.18 to 0.34)*** | 0.26 (0.18 to 0.34)*** |
| Low |  | 0.52 (0.44 to 0.60)*** | 0.52 (0.44 to 0.60)*** | 0.52 (0.44 to 0.60)*** | 0.52 (0.44 to 0.60)*** |
| **Parental control (Ref.Low)** | | | | | |
| Medium |  | 0.07 (-0.01 to 0.15) | 0.07 (-0.01 to 0.15) | 0.07 (-0.01 to 0.45) | 0.06 (-0.01 to 0.15) |
| High |  | 0.41 (0.32 to 0.49)*** | 0.41 (0.32 to 0.50)*** | 0.41 (0.32 to 0.49)*** | 0.41 (0.33 to 0.49)*** |
| **Health behaviours** | | | | | |
| Smoking |  | 0.99 (0.81 to 1.07)*** | 0.94 (0.81 to 1.07)*** | 0.94 (0.81 to 1.07)*** | 0.95 (0.82 to 1.08)*** |
| Alcohol |  | 0.33 (0.25 to 0.41)*** | 0.33 (0.25 to 0.25 to 0.41)*** | 0.33 ( 0.25 to 0.42)*** | 0.34 (0.26 to 0.41)*** |
| **Socio-economic circumstances** | | | | | |
| **Family Affluence Scale (Ref.Least advantaged)** | | | | | |
| Less advantaged |  |  | 0.03 (-0.09 to 0.14) | 0.02 (-0.09 to 0.13) | 0.02 (-0.08 to 0.14) |
| Least disadvantaged |  |  | 0.10 (-0.01 to 0.20) | 0.09 (-0.02 to 0.19) | 0.09 (-0.01 to 0.20) |
| **Combined labour market status and family structure (Ref. 2 parent family, 2 employed)** | | | | | |
| 1 parent family, >=1 employed |  | 0.05 (-0.05 to 0.14) | 0.04 (-0.04 to 0.14) | 0.04 (-0.05 to 0.14) | 0.05 (-0.05 to 0.14) |
| 2 parent family, 0 employed |  | -0.12 (-0.25 to 0.01) | -0.12 (-0.26 to 0.01) | -0.12 (-0.26 to 0.01) | -0.12 (-0.25 to 0.01) |
| 1 parent family, 0 employed |  | 0.01 (-0.12 to 0.15) | 0.01 (-0.12 to 0.15) | 0.01 (-0.12 to 0.15) | 0.01 (-0.12 to 0.15) |
| **IMD (Income domain) (Ref. Least deprived quintile)** | | | | | |
| Least deprived quintile |  |  |  |  |  |
| 2nd |  | 0.11 (0.01 to 0.21)* | 0.11 (0.01 to 0.21)* | 0.11 (0.01 to 0.21)* | 0.11 (0.01 to 0.21)* |
| 3rd |  | 0.11 (0.00 to 0.21)* | 0.11 (0.00 to 0.21)* | 0.11 (0.00 to 0.21)* | 0.11 (0.00 to 0.21)* |
| 4th |  | 0.17 (0.06 to 0.27)** | 0.17 (0.06 to 0.27)** | 0.16 (0.06 to 0.27)** | 0.17 (0.06 to 0.27)** |
| Most deprived quintile |  | 0.19 (0.07 to 0.30)** | 0.19 (0.07 to 0.30)** | 0.18 (0.07 to 0.30)** | 0.19 (0.07 to 0.30)** |
| **Moderation** | | | | | |
| Pollutant*racism |  |  | 0.04 (0.00 to 0.08)* |  | 0.05 (0.01 to 0.10)** |
| Pollutant*ethnicity (Ref. White British) |  |  |  |  |  |
| Black Caribbean |  |  |  | -0.02 (-0.07 to 0.04) | -0.02 (-0.08 to 0.03) |
| Black African |  |  |  | -0.06 (-0.11 to -0.01)* | -0.07 (-0.12 to -0.02)** |
| Indian |  |  |  | -0.03 (-0.10 to 0.03) | -0.04 (-0.10 to 0.02) |
| Pakistani & Bangladeshi |  |  |  | -0.10 (-0.16 to -0.03)** | -0.11 (-0.17 to -0.04)** |
| Others |  |  |  | -0.06 (-0.10 to -0.01)* | -0.07 (-0.12 to -0.02)** |
| Random Effects | | | | | |
| Level 2(Between-child intercept variance) | 1.08 (1.04 to 1.12)*** | 0.89 (0.85 to 0.94)*** | 0.89 (0.85 to 0.93)*** | 0.89 (0.85 to 0.94)*** | 0.89 (0.85 to 0.93)*** |
| Level 1 (occasion) | 1.28 (1.25 to 1.30)*** | 1.25 (1.22 to 1.28)*** | 1.25 (1.22 to 1.29)*** | 1.25 (1.22 to 1.28)*** | 1.25 (1.22 to 1.29)*** |

Note: * p<0.05, **p<0.01, *** p<0.001

Model 1 -adjusted for age, PM _2.5_, and PM _2.5_ * age

Model 2 – Model 1+ racism,sex, ethnicity, maternal mental health problems, parental care, parental control, cigarette smoking, alcohol, family affluence and IMD (income domain).

Model 3-Model 2 + PM _2.5_ * racism

Model 4-Model 2 + PM _2.5_ * ethnicity

Model 5–Model 2+ PM _2.5_*racism+ PM _2.5_ * ethnicity

***Supplementary Table 3:*** *Pooled and interactive effects of NO2 on trajectories of adolescent conduct problems.*

|  | Model 1 | Model 2 | Model 3 | Model 4 | Model 5 |
| --- | --- | --- | --- | --- | --- |
|  | **Coefficient (95% confidence interval)** | **Coefficient (95% confidence interval)** | **Coefficient (95% confidence interval)** | **Coefficient (95% confidence interval)** | **Coefficient (95% confidence interval)** |
| Fixed effects | | | | | |
| **Pollutant** | -0.01 (-0.02 to 0.01) | -0.02 (-0.03 to 0.00)* | -0.02 (-0.04 to 0.00)* | -0.02 (-0.04 to 0.00)* | -0.03 (-0.05 to 0.01)* |
| **Age** | -0.23 (-0.44 to -0.01)* | -0.48 (-0.69 to -0.27)*** | -0.48 (-0.70 to -0.27)*** | -0.48 (-0.70 to -0.27)*** | -0.48 ( -0.69 to -0.27)*** |
| **Pollutant*age** | 0.01 (0.00 to 0.01) | 0.01 (0.00 to 0.01)*** | 0.01 (0.00 to 0.01)** | 0.01 (0.00 to 0.01)** | 0.01 (0.00 to 0.01)** |
| **Racism** |  | 0.36 (0.28 to 0.43)*** | 0.40 (-0.33 to 1.14) | 0.36 (0.28 to 0.43)*** | 0.44 (-0.30 to 1.18) |
| **Female** |  | -0.36 (-0.43 to -0.28)*** | -0.36 (-0.43 to -0.28)*** | -0.36 (-0.43 to -0.28)*** | -0.36 (-0.43 to -0.28)*** |
| **Ethnicity (Ref. White British)** | | | | | |
| Black Caribbean |  | 0.22 (0.09 to 0.35)** | 0.20 (0.06 to 0.15)** | -0.01 (-1.22 to 1.21) | 0.00 (-1.22 to 1.24) |
| Black African |  | 0.13 (0.00 to 0.26) | 0.12 (-0.01 to 0.25) | -0.58 (-1.70 to 0.53) | -0.57 (-1.71 to 0.56) |
| Indian |  | -0.15 (-0.31 to 0.01) | -0.14 (-0.30 to 0.01) | -0.22 (-1.93 to 1.48) | -0.23 (-1.94 to 1.48) |
| Pakistani & Bangladeshi |  | -0.08 (-0.23 to 0.07) | -0.06 (-0.21 to 0.09) | 0.54 (-1.09 to 2.18) | 0.47 (-1.17 to 2.12) |
| Others |  | 0.04 (-0.07 to 0.15) | 0.04 (-0.07 to 0.16) | -0.22 (-1.25 to 0.80) | -0.24 (-1.27 to 0.79) |
| **Maternal mental health problems (Yes)** |  |  | 0.23 (0.07 to 0.39)** | 0.23 (0.07 to 0.39)** |  |
| **Parental care (Ref.High)** | | | | | |
| Medium |  | 0.27 (0.19 to 0.35)*** | 0.27 (0.19 to 0.35)*** | 0.27 (0.19 to 0.35)*** | 0.27 (0.19 to 0.35)*** |
| Low |  | 0.53 (0.44 to 0.61)*** | 0.52 (0.44 to 0.61)*** | 0.52 (0.44 to 0.61)*** | 0.53 (0.44 to 0.61)*** |
| **Parental control (Ref.Low)** | | | | | |
| Medium |  | 0.06 (-0.01 to 0.15) | 0.07 (-0.01 to 0.15) | 0.07 (-0.01 to 0.15) | 0.07 (-0.01 to 0.15) |
| High |  | 0.40 (0.32 to 0.49)*** | 0.40 (0.32 to 0.49)*** | 0.40 (0.32 to 0.49)*** | 0.41 (0.32 to 0.49)*** |
| **Health behaviours** |  |  |  |  |  |
| Smoking |  | 0.93 (0.80 to 1.06)*** | 0.93 (0.80 to 1.06)*** | 0.93 (0.80 to 1.06)*** | 0.93 (0.80 to 1.06)*** |
| Alcohol |  | 0.34 (0.26 to 0.41)*** | 0.33 (0.26 to 0.40)*** | 0.33 (0.26 to 0.40)*** | 0.33 (0.25 to 0.41)*** |
| **Socio-economic circumstances** | | | | | |
| **Family Affluence Scale (Ref.Least advantaged)** | | | | | |
| Less advantaged |  | 0.03 (-0.08 to 0.14) | 0.03 (-0.08 to 0.14) | 0.03 (-0.08 to 0.14) | 0.03 (-0.09 to 0.14) |
| Least disadvantaged |  | 0.10 (0.00 to 0.24) | 0.10 (0.00 to 0.24) | 0.10 (0.00 to 0.24) | 0.09 (-0.01 to 0.20) |
| **Combined labour market status and family structure (Ref. 2 parent family, 2 employed)** | | | | | |
| 1 parent family, >=1 employed |  | 0.05 (-0.04 to 0.15) | 0.05 (-0.04 to 0.15) | 0.05 (-0.04 to 0.15) | 0.05 (-0.04 to 0.15) |
| 2 parent family, 0 employed |  | -0.12 (-0.25 to 0.01) | -0.12 (-0.25 to 0.01) | -0.12 (-0.25 to 0.01) | -0.12 (-0.25 to 0.02) |
| 1 parent family, 0 employed |  | 0.01 (-0.13 to 0.15) | 0.01 (-0.13 to 0.15) | 0.01 (-0.13 to 0.15) | 0.01 (-0.12 to 0.15) |
| **IMD (Income domain) (Ref. Least deprived quintile)** | | | | | |
| 2nd |  | 0.12 (0.02 to 0.22)* | 0.12 (0.02 to 0.22)* | 0.12 (0.02 to 0.22)* | 0.12 (0.02 to 0.22)* |
| 3rd |  | 0.11 (0.02 to 0.22)* | 0.11 (0.02 to 0.22)* | 0.11 (0.02 to 0.22)* | 0.12 (0.02 to 0.22)* |
| 4th |  | 0.18 (0.07 to 0.29)** | 0.18 (0.07 to 0.29)** | 0.18 (0.07 to 0.29)** | 0.18 (0.07 to 0.29)** |
| Most deprived quintile |  | 0.19 (0.08 to 0.32)** | 0.20 (0.08 to 0.31)** | 0.20 (0.08 to 0.31)** | 0.20 (0.08 to 0.32)** |
| **Moderation** | | | | | |
| Pollutant*racism |  |  | 0.00 (-0.02 to 0.02) |  | 0.00 (-0.02 to 0.02) |
| Pollutant*ethnicity (Ref. White British) |  |  |  |  |  |
| Black Caribbean |  |  |  | 0.00 (-0.02 to 0.03) | 0.00 (-0.02 to 0.03) |
| Black African |  |  |  | 0.02 (-0.01 to 0.04) | 0.02 (-0.025 to 0.03) |
| Indian |  |  |  | 0.00 (-0.04 to 0.04) | 0.00 (-0.04 to 0.04) |
| Pakistani & Bangladeshi |  |  |  | -0.01 (-0.06 to 0.02) | -0.01 (-0.05 to 0.03) |
| Others |  |  |  | 0.00 (-0.02 to 0.03) | 0.01 (-0.02 to 0.032) |
| Random Effects | | | | | |
| Level 2 (Between-child intercept variance) | 1.08 (1.03 to 1.15)*** | 0.89 (0.85 to 0.93)*** | 0.89 (0.85 to 0.93)*** | 0.89 (0.85 to 0.93)*** | 0.89 (0.85 to 0.93)*** |
| Level 1 (occasion) | 1.28 (1.25 to 1.30)*** | 1.26 (1.23 to 1.28)*** | 1.26 (1.23 to 1.28)*** | 1.26 (1.23 to 1.28)*** | 1.26 (1.23 to 1.28)*** |

Note: * p<0.05, **p<0.01, *** p<0.001

Model 1 -adjusted for age, NO_2_, and NO_2_* age

Model 2 – Model 1+ racism, sex, ethnicity, maternal mental health problems, parental care, parental control, cigarette smoking, alcohol, family affluence and IMD (income domain).

Model 3-Model 2 + NO_2_ * racism

Model 4-Model 2 + NO_2_ * ethnicity

Model 5 –Model 2 + NO_2_*racism+ NO_2_* ethnicity

***Supplementary Table 4:*** *Sensitivity Analysis of Models 2 and 6 exploring the Association Between Annualized Average Levels of PM_2.5_ and trajectories of adolescent conduct problems using complete cases (N=2551)*

|  | Model 2 | Model 5 |
| --- | --- | --- |
|  | **Coefficient (95% confidence interval)** | **Coefficient (95% confidence interval)** |
| Fixed effects | | |
| **Pollutant** | -0.12 (-0.21 to -0.03)** | -0.15 (-0.26 to -0.04)** |
| **Age** | -1.32 (-1.97 to -0.67)*** | -1.30 (-1.95 to -0.63)*** |
| **Pollutant*age** | 0.07 (0.03 to 0.10)*** | 0.06 (0.02 to 0.10)** |
| **Racism** | 0.34 (0.22 to 0.46)*** | -0.66 (-1.82 to 0.50) |
| **Female** | -0.27 (-0.38 to -0.15)*** | -0.27 (-0.38 to -0.15)*** |
| **Ethnicity (Ref. White British)** | | |
| Black Caribbean | 0.17 (0.00 to 0.34) | 0.43 (-1.03 to 1.88) |
| Black African | 0.12 (-0.06 to 0.29) | -0.14 (-1.54 to 1.26) |
| Indian | -0.22 (-0.42 to -0.02)* | -0.44 (-2.00 to 1.12) |
| Pakistani & Bangladeshi | 0.01 (-0.19 to 0.21) | 0.71 (-0.91 to 2.32) |
| Others | 0.18 (-0.05 to 0.41) | 0.44 (-2.22 to 3.10) |
| **Moderation** | | |
| Pollutant*female |  | 0.02 (-0.04 to 0.08) |
| Pollutant*racism |  | 0.06 (-0.01 to 0.12) |
| Pollutant*ethnicity (Ref. White British) |  |  |
| Black Caribbean |  | -0.02 (-0.10 to 0.07) |
| Black African |  | 0.01 (-0.06 to 0.09) |
| Indian |  | 0.01 (-0.07 to 0.10) |
| Pakistani & Bangladeshi |  | -0.04 (-0.13 to 0.05) |
| Others |  | -0.01 (-0.17 to 0.14) |
| Random Effects | | |
| Level 2(Between-child intercept variance) | 0.92 (0.84 to 1.01)*** | 0.92 (0.84 to 1.01)*** |
| Level 1 (occasion) | 1.18 (1.12 to 1.24)*** | 1.18 (1.12 to 1.24)*** |

Note: * p<0.05, **p<0.01, *** p<0.001

Model 2 – Model 1+ racism, sex, ethnicity, maternal mental health problems, parental care, parental control, cigarette smoking, alcohol, family affluence and IMD (income domain).

Model 5: Adjusted for PM _2.5_, age, PM _2.5_ * age, racism, PM _2.5_* racism, ,ethnicity, PM _2.5_ * ethnicity, sex, maternal mental health problems, parental care, parental control, cigarette smoking, alcohol, family affluence and IMD (income domain).

***Supplementary Table 5:*** *Sensitivity Analysis of 2-Pollutant Model of the Association Between Annualized Average Levels of PM_2.5_ and NO_2_ and trajectories of adolescent conduct problems.*

|  | **Model 5** |
| --- | --- |
|  | **Coefficient (95% confidence interval)** |
| Fixed effects | |
| **PM 2.5** | -0.08 (-0.16 to 0.00)* |
| **NO2** | 0.00 (-0.02 to 0.02) |
| **Age** | -1.20 (-1.59 to -0.81) |
| **PM 2.5*age** | 0.07 (0.04 to 0.09)*** |
| **NO2 * age** | 0.00 (-0.01 to 0.00) |
| **Female** | -0.36 (-0.43 to -0.28)*** |
| **Racism** | 0.37 (0.29 to 0.44)*** |
| **Ethnicity (Ref. White British)** | |
| Black Caribbean | 0.68 (-0.28 to 1.65) |
| Black African | 1.39 (0.45 to 2.32)** |
| Indian | 0.60 (-0.52 to 1.71) |
| Pakistani & Bangladeshi | 1.84 (0.74 to 2.94)** |
| Others | 1.28 (0.44 to 2.12)** |
| **Moderation** |  |
| **Pollutant*racism** | 0.06 (0.01 to 0.10)** |
| **Pollutant*ethnicity (Ref. White British)** | |
| Black Caribbean | -0.03 (-0.08 to 0.03) |
| Black African | -0.07 (-0.12 to -0.02)** |
| Indian | -0.04 (-0.10 to 0.02) |
| Pakistani & Bangladeshi | -0.11 (-0.16 to -0.05)** |
| Others | -0.07 (-0.12 to -0.02)** |
| Random Effects | |
| Level 2 (Between-child intercept variance) | 0.89 (0.85 to 0.93) |
| Level 1 (occasion) | 1.25 (1.22 to 1.29) |

Note: * p<0.05, **p<0.01, *** p<0.001

Model 5: Adjusted for PM _2.5_, NO_2_, age, PM _2.5_ * age, NO_2_ * age, racism, PM _2.5_* racism, ethnicity, PM _2.5_ * ethnicity, sex ,maternal mental health problems, parental care, parental control, cigarette smoking, alcohol, family affluence and IMD (income domain).


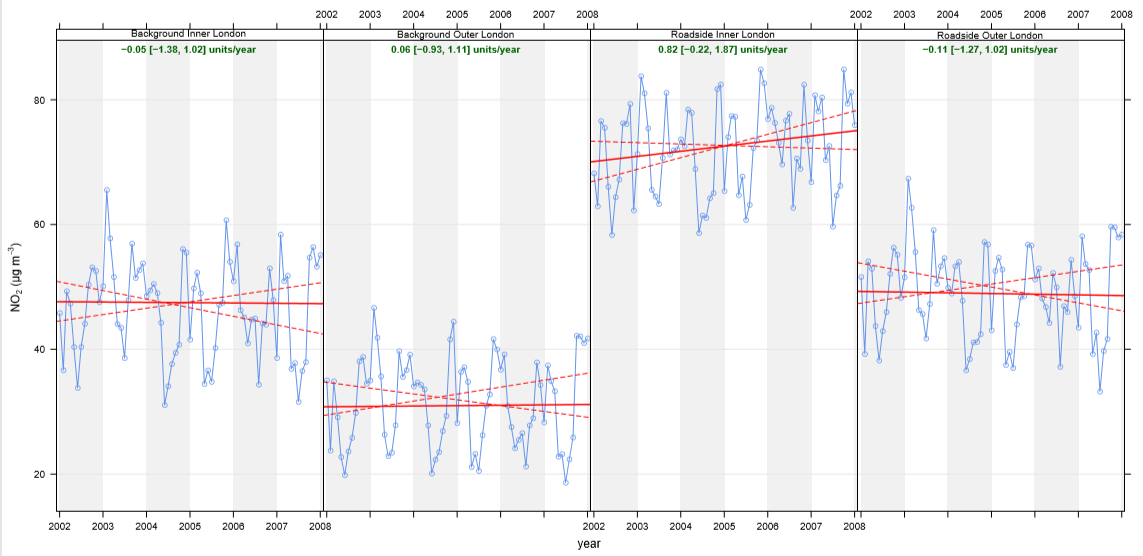

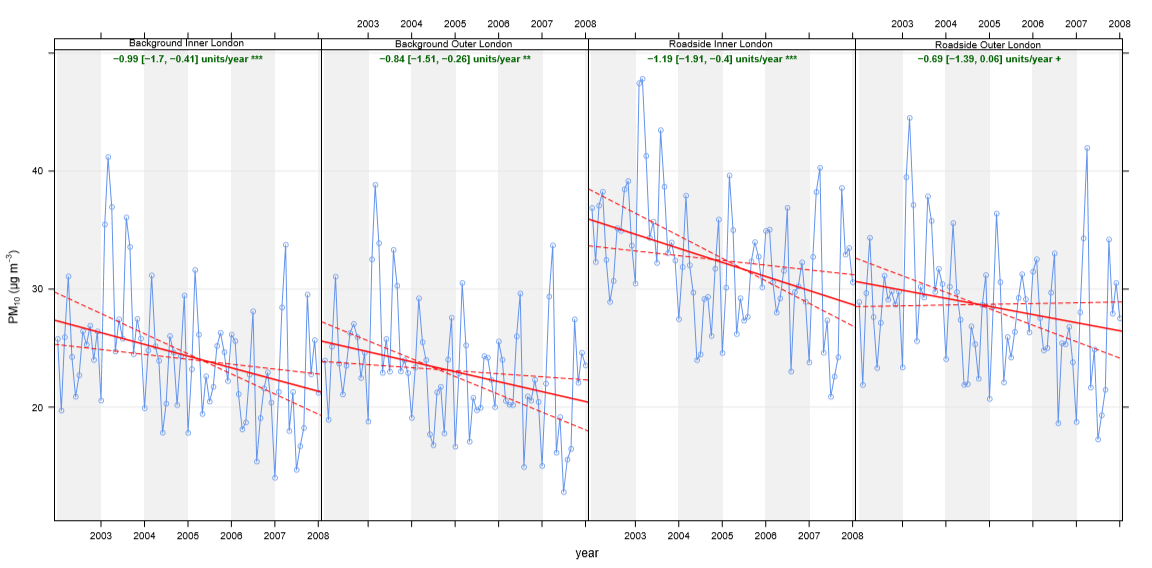

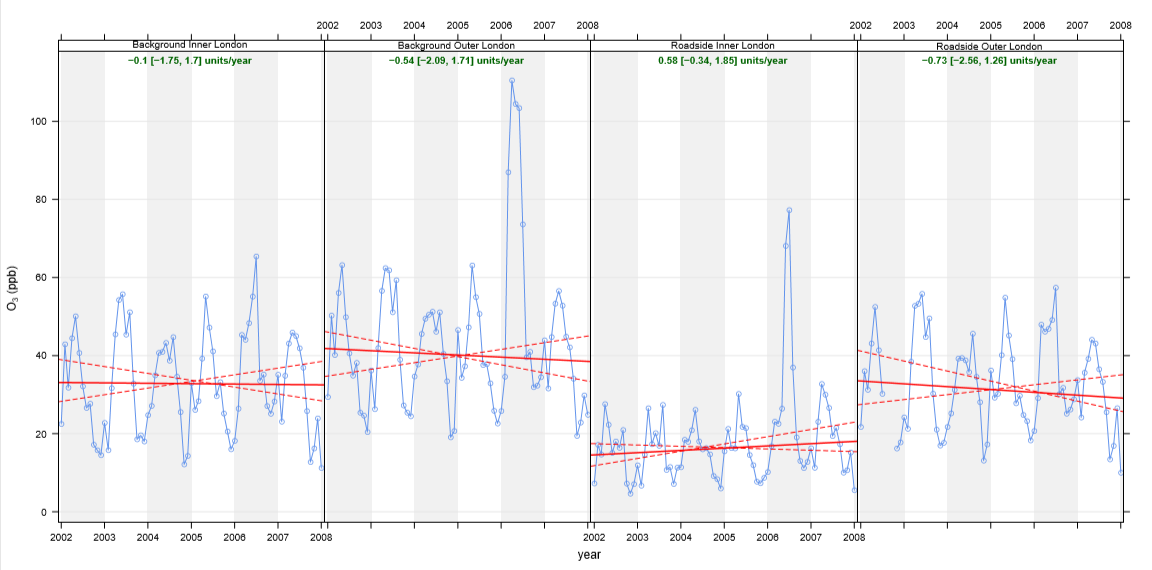


**Supplementary Figure 1:** Changes in monthly average concentrations of NO_2_, PM_10_ and O^3^ between 2002 and 2008 using sites classified as inner and outer London roadside (RS) and background (BG) locations. For NO_2_ the data from the following number of sites were averaged over the period: inner London RS (n=6), outer London RS (n=7), inner London BG (n=4), outer London BG (n=9). For PM_10_ the equivalent site number were n=7, 10, 3 and 5, and for O_3_: n=3, 3, 4 and 5. Insufficient sites monitored PM_2.5_ at the beginning of the study period and therefore trends are not shown. In each panel the rate of change in pollutant concentration is illustrated (mean with 95% confidence interval), as µg/m^3^ per year.’


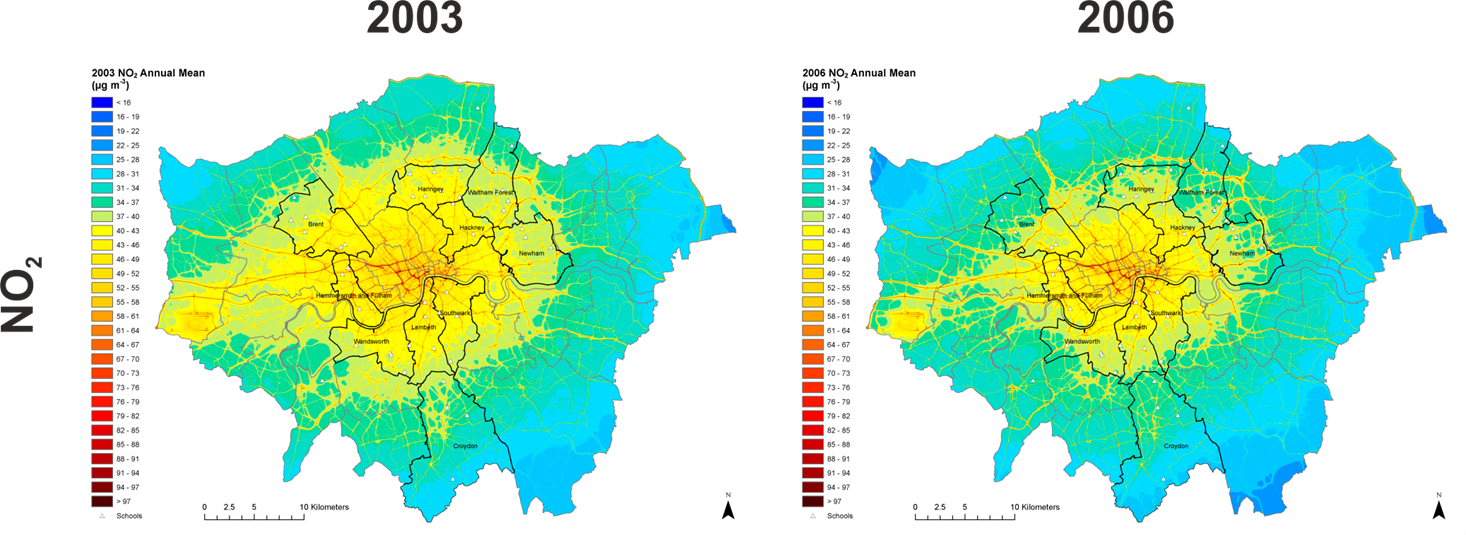

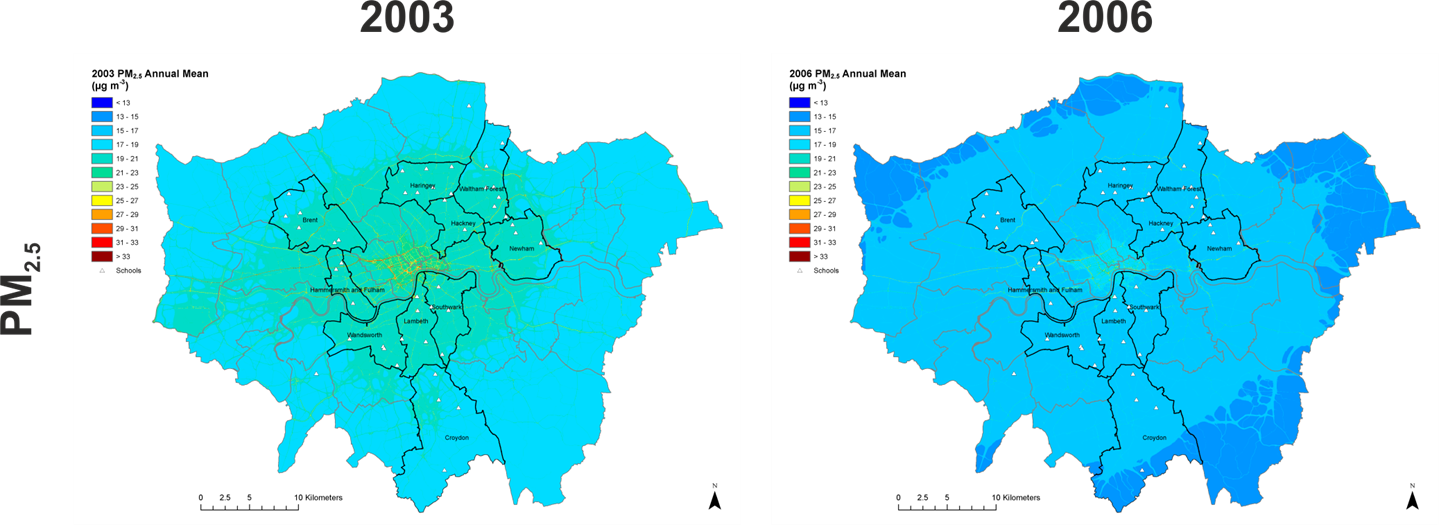


**Supplementary Figure 2**: Modelled Greater London concentrations (20m^2^ resolution) for O_3_, NO_2_, PM_10_ and PM_2.5_ for representative years 2003 and 2006. The ten boroughs in which the study took place are highlighted together with the locations of the participating schools (open triangles).
